# pH-dependent virucidal effects of weak acids against pathogenic viruses

**DOI:** 10.1186/s41182-023-00573-1

**Published:** 2024-01-12

**Authors:** Weiyin Hu, Hiroshi Shimoda, Yoshihiro Tsuchiya, Mikiya Kishi, Daisuke Hayasaka

**Affiliations:** 1 Laboratory of Veterinary Microbiology, Joint Graduate School of Veterinary Medicine, Yamaguchi University, 1677-1 Yoshida, Yamaguchi, 753-8511 Japan; 2 Central Research Institute, Mizkan Holdings Co., Ltd., 2-6 Nakamura-Cho, Handa-Shi, Aichi, 475-8585 Japan

**Keywords:** Acetic acid, Oxalic acid, Citric acid, Weak acids’ virucidal effects, Influenza virus, SARS-CoV-2, Feline calicivirus, pH, Vinegar

## Abstract

**Background:**

Weak acids, such as acetic acid, show virucidal effects against viruses, and disinfectants are considered effective virucidal agents possibly because of their low pH, depending on the proton concentration. This study aimed to evaluate the efficacy of different weak acids (acetic, oxalic, and citric acids) and eligible vinegars under different pH conditions by comparing their inactivation efficacies against enveloped and non-enveloped viruses.

**Methods:**

Acetic, oxalic, and citric acids were adjusted to pH values of 2, 4 and 6, respectively. They were also diluted from 1 M to 0.001 M with distilled water. Enveloped influenza A virus (FulV) and severe acute respiratory syndrome coronavirus 2 (SARS-CoV-2) and non-enveloped feline calicivirus (FCV) were treated with adjusted weak acids for up to 30 min. These viruses were also reacted with white distilled vinegar (WDV) and grain-flavored distilled vinegar (GV) for up to 30 min. Infectious viral titers after the reactions were expressed as plaque-forming units per mL.

**Results:**

Acetic acid showed virucidal effects against FulV at pH 4, whereas citric and oxalic acids did not. Acetic and citric acids inactivated SARS-CoV-2 at pH 2, whereas oxalic acid did not. All acids showed virucidal effects against FVC at pH 2; however, not at pH 4. The virucidal effects of the serially diluted weak acids were also reflected in the pH-dependent results. WDV and GV significantly reduced FulV titers after 1 min. SARS-CoV-2 was also susceptible to the virucidal effects of WDV and GV; however, the incubation period was extended to 30 min. In contrast, WDV and GV did not significantly inactivate FCV.

**Conclusions:**

The inactivation efficacy of weak acids is different even under the same pH conditions, suggesting that the virucidal effect of weak acids is not simply determined by pH, but that additional factors may also influence these effects. Moreover, eligible vinegars, the main component of which is acetic acid, may be potential sanitizers for some enveloped viruses, such as FulV, in the domestic environment.

**Supplementary Information:**

The online version contains supplementary material available at 10.1186/s41182-023-00573-1.

## Background

Viral infectious diseases, such as coronavirus disease 2019 [1–3], influenza [4–6] and norovirus gastroenteritis [7, 8], are major domestic public health concerns. These diseases are highly contagious, which makes the inactivation of these viruses a matter of utmost priority in preventing the spread of infections. Inactivation of these viruses is of the utmost priority in preventing the spread of infections.

Disinfectants, such as ethanol [9, 10] and sodium hypochlorite [11, 12], are generally used as potent virucidal agents domestically. Weak acids, such as acetic acid, are also effective against pathogenic viruses. Acetic acid exerts virucidal effects against enveloped viruses, such as vaccinia virus, African swine fever virus, influenza virus (FulV), and severe acute respiratory syndrome coronavirus (SARS-CoV) [13–17]. In addition, vinegar, the main component of which is acetic acid, shows efficient virucidal activity against SARS-CoV-2 [15, 18, 19]. It is a natural product that is harmless to the human body [20–22]; thus, the use of edible vinegars as cleaning and sanitizing agents is promising domestically [23–25].

The virucidal effect of acids is considered to be due to the low pH. Therefore, any weak acid seems to exert similar inactivation effects under the same pH conditions. However, the effective pH of weak acids has not yet been fully elucidated. In this study, we aimed to compare the inactivation effects of acetic, oxalic, and citric acids against two enveloped viruses, SARS-CoV-2 and FulV, and a non-enveloped virus, feline calicivirus (FCV), under different pH conditions. We further demonstrated the virucidal effects of eligible vinegars. These observations provide useful information regarding the appropriate conditions for weak acids against pathogenic viruses.

## Methods

### Viruses and cells

SARS-CoV-2 (2019-nCoV/Japan/AI/I-004/2020, National Institute of Infectious Diseases [NIID] strain) was kindly provided by the NIID (Tokyo, Japan). FulV A/H1N1 was provided by the Yamaguchi Prefecture Institute of Public Health and Environment (Yamaguchi, Japan). FCV was obtained from the American Type Culture Collection and was used as a surrogate for norovirus.

FulV, SARS-CoV-2, and FCV were propagated in Madin–Darby canine kidney (MDCK), VeroE6/TMPRSS2 (JCRB 1819), and Crandell–Rees Feline Kidney (CRFK) cells, respectively. VeroE6/TMPRSS2 was kindly provided by the NIID. These cells were propagated in Dulbecco’s modified Eagle’s minimum essential medium (DMEM; Thermo Fisher Scientific Inc., Waltham, Massachusetts, USA) supplemented with 10% heat-inactivated fetal bovine serum (FBS) (JR Scientific, Woodland, CA, USA), 100 U/ml penicillin, and 100 µg/ml streptomycin (Life Technologies, Carlsbad, CA, USA) and maintained at 37 °C in 5% CO_2_.

Virus-infected cells were incubated at 37 °C in 5% CO_2_ in DMEM supplemented with 2% FBS. The supernatants of these growth media were stored at – 80 °C as stock sources of viruses. Stocked virus titers were 6.33 × 10^6^ to 3.57 × 10^7^ plaque-forming units (pfu)/ml of FulV, 5.4 × 10^6^ to 6.93 × 10^7^ pfu/ml of SARS-CoV-2, and 1.04 × 10^7^ to 1.57 × 10^8^ pfu/ml of FCV in each experiment.

Experiments using infectious SARS-CoV-2 were performed in a biosafety level (BSL)-3 laboratory at Yamaguchi University following standard BSL-3 guidelines. FulV and FCV experiments were performed in a BSL-2 laboratory.

### Plaque-forming assay

The infectious viral titers of SARS-CoV-2, FulV, and FCV were determined using a plaque-forming assay. Briefly, each type of cell was grown in 12-well plates until confluent; subsequently, serially diluted viruses were inoculated (100 μl/well). SARS-CoV-2 and FCV were diluted in DMEM supplemented with 2% FBS, and FulV was diluted in DMEM supplemented with 0.001% trypsin (trypsin from Hog pancreas 1:250; NACALAI TESQUE. Tokyo, Japan) and 0.2% bovine serum albumin (BSA) (SIGMA, USA). After incubation at 37 °C for 90 min, the inoculum was removed, and the cells were washed with DMEM. Subsequently, 0.8% agarose (SeaPlaque GTG Agarose, Lonza, Rockland, ME, USA) in DMEM supplemented with 10% FBS or DMEM supplemented with 0.001% trypsin and 0.2% BSA was added to each well. The plates were incubated at 37 ℃ until visible plaques appeared. Plaque formation was observed at 36 h post-infection (pi) for SARS-CoV-2, 48 h pi for FulV, and 24 h pi for FCV. We confirmed that trypsin in the media did not influence pH of reaction samples and plaque formations for FulV (data not shown). To retrieve the plate, the cells were fixed with 10% formalin and thoroughly stained with 1% crystal violate (FUJIFILM WAKO, Osaka, Japan). The viral titers were expressed as pfu/ml.

### Weak acids

In total, 1 M acetic acid (FUJIFILM, conduct log: 017–00256), 1 M oxalic acid (WAKO, conduct log: 150–00455), and 1 M citric acid (FUJIFILM, conduct log: 038–06925) were diluted using sterilized water from the original acid solutions. To adjust the acetic acid to pH 2, 4, and 6, 0.12-, 0.2-, and 1-time volumes of 1 M sodium hydroxide (NaOH) were added to 1 M acetic acid, respectively. Correspondingly, 1.08-, 1.5-, and 1.9-time volumes of 1 M NaOH and 0.14-, 1.3-, and 2.59-time volumes of 1 M NaOH were added to 1 M oxalic acid and 1 M citric acid, respectively, to adjust the pH to 2, 4, and 6. Each 1 M acid was serially diluted to 1, 0.1, 0.01, and 0.001 M with sterilized water.

### Inactivation of viruses by weak acids

A total of 900 μl of each adjusted weak acid was mixed with 100 μl of each viral solution in 2% FBS-supplemented DMEM at a 9:1 ratio and reacted for 1, 10 and 30 min at room temperature. The pH of the reaction mixtures did not significantly change from the original adjusted values (Table 1).

**Table 1:** pH in the reaction mixtures of pH-dependent acids and 2%FBS DMEM

Acid	Original adjusted pH	pH in the reaction mixture^*^
Acetate	2.07	2.04 ± 0.12
	4	4.09 ± 0.01
	6.03	6.03 ± 0.09
Citrate	2.05	2.24 ± 0.02
	4.04	4.24 ± 0.10
	6	6.00 ± 0.15
Oxalate	2.02	2.02 ± 0.05
	3.96	4.09 ± 0.13
	6.01	6.77 ± 0.02

pH in original adjusted weak acids was measured, following to measure pH in mixture in mixture in triplicate

^*^Average value in triplicate ± 95% confidence interval

The pH values in the reaction mixtures of molarity-dependent mixtures were 2.39–7.35 in acetic acid, 1.27–6.41 in citric acid, and 0.63–6.79 in oxalic acid (Table 2).

**Table 2:** pH in the reaction mixtures of molarity-dependent acids and 2%FBS DMEM

Acid	Molarity of acid	Original adjusted pH	pH in the reaction mixture^*^
Acetate	1 M	2.31	2.39 ± 0.090
	0.1 M	2.73	3.25 ± 0.100
	0.01 M	3.31	4.46 ± 0.258
	0.001 M	3.94	7.35 ± 0.259
Citrate	1 M	1.25	1.27 ± 0.094
	0.1 M	1.85	2.02 ± 0.112
	0.01 M	2.46	2.94 ± 0.169
	0.001 M	3.2	6.41 ± 0.080
Oxalate	1 M	0.59	0.63 ± 0.100
	0.1 M	1.17	1.20 ± 0.000
	0.01 M	1.97	2.25 ± 0.029
	0.001 M	2.95	6.79 ± 0.305

pH in original adjusted weak acids was measured, following to measure pH in mixture in mixture in triplicate

^*^Average value in triplicate ± 95% confidence interval

The reacted solutions were immediately diluted with 2% FBS-supplemented DMEM, and viral titers were determined. Notably, pH values of the weak acid- and edible vinegar-reacted solutions for plaque assays were confirmed (Additional files 1, 2, 3). Cell viabilities of each cell for plaque assay were also evaluated using XTT cell viability assay kit (Biotium, conduct log: 30007, CA, USA) (Additional files 4, 5). From these data, more than 1:100 dilution of any acids did not induce cell damages, indicating that the detection limit of viral titers was 10^3^ pfu/ml for each virus. The effectiveness of the weak acids was determined based on viral loads below the detection limit.

### Vinegars

White distilled vinegar (WDV) and grain-flavored distilled vinegar (GV) containing 4% acetic acid were provided by Mizkan Holdings Co., Ltd., Aichi, Japan. In total, 100 μl of each viral solution was mixed with 900 μl of WDV, GV, and 4% acetic acid for 1, 5, and 30 min, respectively, at room temperature. Subsequently, the samples were immediately diluted with DMEM containing 2% FBS and were applied to cultured cells for titration using the plaque-forming assay. The effectiveness of the weak acids was determined based on viral loads below the detection limit. Notably, > 1:100 dilutions did not cause any cell damage, indicating that the detection limit of the virus titer was 10^3^ pfu/ml.

## Results

### pH-dependent virucidal effects of weak acids

**Fig. 1:**
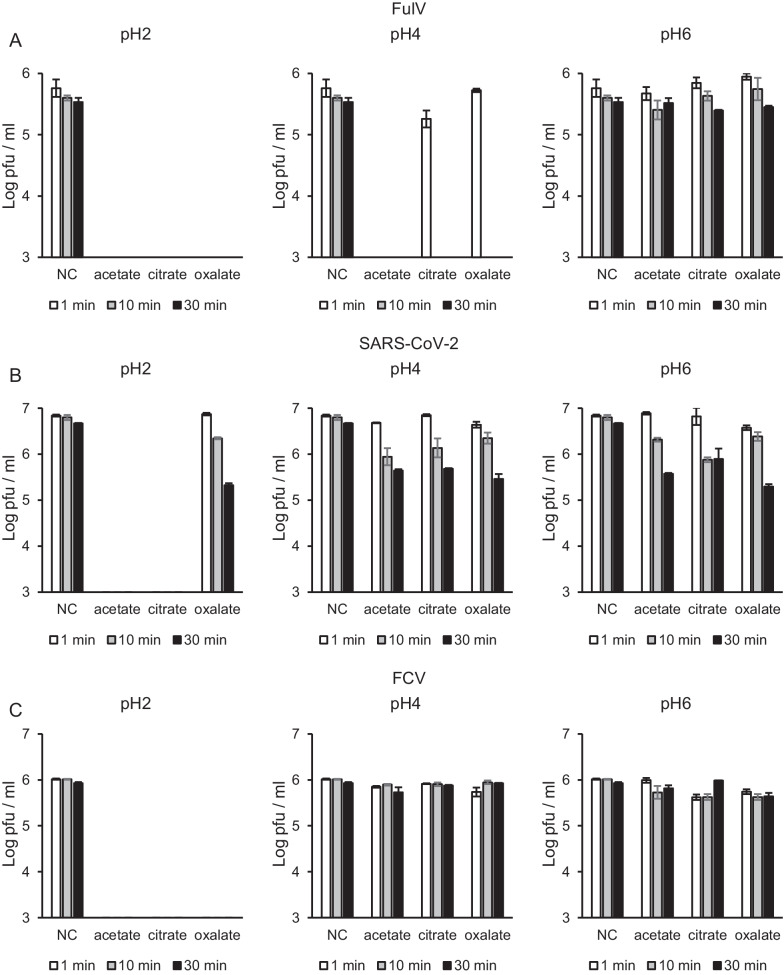
pH-dependent virucidal effects of acetate, oxalate, and citrate against FulV, SARS-CoV-2, and FCV. Acetate, oxalate, and citrate adjusted at pH 2, 4, and 6 were mixed with each virus for 1, 10 and 30 min. Negative control (NC) was incubated with distilled water instead of weak acids. Infectious viral titers (pfu/ml) of FulV (**A**), SARS-CoV-2 (**B**), and FCV (**C**) were determined. Error bars indicate the standard error. Detection limit was set to 10^3^ pfu/ml. Each viral inactivation was accessed in triplicate

Against FulV, acetic acid caused significant reductions in viral titers below the detection limit at pH 2 and pH 4; however, not at pH 6 (Fig. 1A). In contrast, citric and oxalic acids substantially reduced the titers at pH 2 for 1 min and at pH 4 after 10 min; however, it did not show virucidal effects at pH 4 for 1 min and at pH 6 (Fig. 1A). These observations suggest that in short reaction time for 1 min acetic acid shows potentially stronger effects against FulV than oxalic and citric acids in under the same pH conditions.

Interestingly, the effects of weak acids on SARS-CoV-2 were different from those of FulV, although both are enveloped viruses. Acetic and citric acids caused a significant reduction in viral titers of SARS-CoV-2 at pH 2, whereas oxalic acid did not (Fig. 1B). None of the acids exhibited a significant reduction to under detection limits in viral titers at pH 4 and 6, although viral titers were slightly reduced up to 30 min incubation in each reaction (Fig. 1B). These results indicate that the virucidal effect of oxalic acid is limited to SARS-CoV-2 when compared with that of acetic and oxalic acids under low pH (pH 2) conditions.

Acetic, oxalic, and citric acids significantly reduced virus titers of FCV to below the detection limit at pH 2; however, the same level of reduction was not observed at pH 4 and 6 (Fig. 1C), indicating that the inactivation effects against FCV are not significantly different among acetic, citric, and oxalic acids.

These findings suggest that the inactivation efficacy of weak acids differs for different viruses under the same pH conditions. Acetic acid has a potentially greater effect compared with citric acid and oxalic acids.

### Concentration-dependent virucidal effects of weak acids

**Fig. 2:**
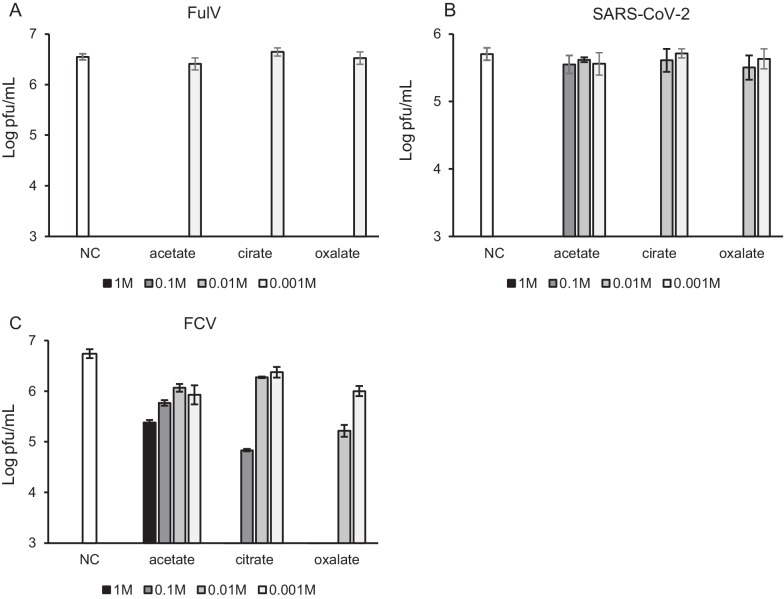
Concentration-dependent virucidal effects of acetate, oxalate, and citrate against FulV, SARS-CoV-2 and FCV. In total, 1, 0.1, 0.01, and 0.001 M of acetate, oxalate, and citrate were mixed with each virus for 1 min. NC was incubated with distilled water instead of weak acids. Infectious viral titers (pfu/ml) of FulV (**a**), SARS-CoV-2 (**b**), and FCV (**c**) were determined. Error bars indicate the standard error. Detection limit was set to 10^3^ pfu/ml. Each viral inactivation was accessed in triplicate

The virucidal effects of each acid, depending on serial concentrations of 0.001–1 M, were also examined (Table 2). In total, 0.01–1 M acetic acid (pH 2.39–4.46), citric acid (pH 1.27–2.94), and oxalic acid (pH 0.63–2.25) showed significant reductions of FulV titers (Fig. 2A), whereas 0.001 M acetic acid (pH 7.35), citric acid (pH 6.41), and oxalic acid (pH 6.79) did not. These results corresponded to the pH-dependent effects shown in Fig. 1A.

Against SARS-CoV-2, 1 M acetic acid (pH 2.39) showed a significant reduction in viral titers; however, 0.001–0.1 M (pH 3.25–7.35) did not (Fig. 2B). Moreover, 1 M (pH 1.27) and 0.1 M (pH 2.02) citric acid exhibited significant inactivation of viral titers; however, 0.01 M (pH 2.94) and 0.001 M (pH 6.41) did not (Fig. 2B). In addition, 1 M (pH 0.63) and 0.1 M (pH 1.20) oxalic acid showed significant reduction of viral titers; however, 0.01 M (pH 2.25) and 0.001 M (pH 6.79) did not (Fig. 2B). These effects corresponded to the pH-dependent results shown in Fig. 1B.

Against FCV, 1–0.001 M acetic acid (pH 2.39–7.35) did not show significant inactivation (Fig. 2c). Moreover, 1 M (pH 1.27) citric acid showed virucidal activity; however, 0.1–0.001 M citric acid (pH 2.02–6.41) did not (Fig. 2C). Furthermore, 1 M (pH 0.63) and 0.1 M (pH 1.20) oxalic acid significantly inactivated FCV; however, 0.01 M (pH 2.25) and 0.001 M (pH 6.79) did not (Fig. 2C). These results mostly corresponded to the pH-dependent results shown in Fig. 1C, although the effects of approximately pH 2, including 0.1 M citric acid (pH 2.02) and 0.01 M oxalic acid (pH 2.25), were different from the results of Fig. 1C. These differences may be due to the dependence of the efficacy on the acid concentration.

These observations suggest that pH mainly influences the virucidal activity of each weak acid. Taken together, acetic acid can inactivate FulV at approximately pH 4; however, citric and oxalic acids require pH lower than 4 for the same efficacy. In addition, acetic and citric acids can inactivate SARS-CoV-2 at approximately pH 2, whereas oxalic acid requires a pH < 2. These findings suggest that the virucidal activity of weak acids does not simply depend on proton concentration, and other factors of each weak acid may also contribute significantly to these effects.

### Virucidal effects of eligible vinegars

Our observations imply that acetic acid effectively reduces the infectious virus concentrations under higher pH conditions compared with other weak acids. Therefore, we examined the virucidal activity of vinegars containing acetic acid as the main component.

**Fig. 3:**
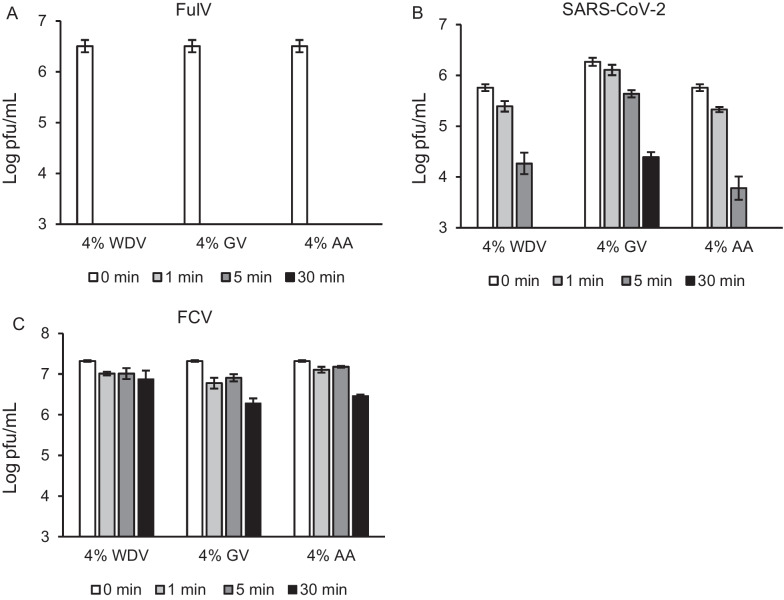
Virucidal effects of eligible vinegars. In total, 4% white distilled vinegar (WDV), 4% grain-flavored distilled vinegar (GV), and 4% acetic acid (AA) were mixed with each virus for 0 (control), 1, 5, and 30 min. Infectious viral titers (pfu/ml) of FulV (**a**), SARS-CoV-2 (**b**), and FCV (**c**) were determined. Error bars indicate the standard error. Detection limit was set to 10^3^ pfu/ml. Each viral inactivation was accessed in triplicate

Similar to 4% acetic acid (pH 2.72), 4% WDV (pH 2.77) and 4% GV (pH 2.79) exhibited significant reductions in FulV titers below the detection limit immediately after 1 min (Fig. 3A). These results reflect the pH-dependent effects of acetic acid on the reaction solutions.

SARS-CoV-2 was also susceptible to the virucidal effects of 4% WDV and 4% acetic acid; however, incubation periods below the detection limits were extended up to 30 min (Fig. 3B). Moreover, 4% GV did not reach the detection limit after 30 min (Fig. 3B). These observations also reflect the pH-dependent effects of acetic acid.

In contrast, 4% WDV (pH 2.77), 4% GV (pH 2.79), and 4% acetic acid (pH 2.72) did not significantly inactivate FCV (Fig. 3C). These results are consistent with those of the adjusted 1 M acetic acid (pH 2.51) shown in Fig. 2C.

These observations suggest that eligible vinegars may be potential sanitizers for some enveloped viruses, such as FulV, in the domestic environment; however, these effects may be limited to enveloped SARS-CoV-2 and non-enveloped viruses.

## Discussion

In general, virucidal activity of acids is due to their low pH, which depends on the molar concentration of protons. Therefore, a higher molar proton concentration is believed to be the key determinant, regardless of the type of acid. Interestingly, our results suggest that there are cases in which inactivation efficacy varies between different types of weak acids, even under the same pH conditions. For example, acetic acid at pH 4 inactivated FulV, whereas citric and oxalic acids did not. Moreover, pH 2 acetic and citric acids showed significant virucidal effects against SARS-CoV-2, whereas oxalic acid did not. These observations suggest that the virucidal effect of weak acids is not simply determined by the pH, and additional factors, such as chemical structure of the acid and other factors, may influence these effects. For example, citric acid and oxalic acid are dicarboxylic acid, and this chemical structure may influence the different effects from acetic acid. Further studies will be required to identify the mechanism of inactivation effects due to different weak acids. In addition, examination of the morphological changes of inactivated viruses using a cryo-electron microscope will provide valuable insights into for understanding the inactivation mechanism by weak acids.

Our results showed that acetic acid was more effective than oxalic and citric acids against the enveloped viruses FulV and SARS-CoV-2 at higher pH. Interestingly, acetic acid induces unique physiological responses in mammalian cells when compared with other types of acids [26], suggesting that acetic acid may penetrate the lipid bilayer of the cell membrane. Therefore, acetic acid may cause physiological changes in the viral envelope and is a potentially effective disinfectant.

Acetic acid is the primary component of vinegar. Edible vinegar is produced by fermentation of plant-based products and is harmless to the human body [21]. It is generally used as a condiment domestically. Vinegar has virucidal effects against SARS-CoV-2 [18]. In this study, 4% WDV and 4% GV showed virucidal efficacy against SARS-CoV-2 and higher effectiveness against FulV. However, these inactivation effects were limited against FCV. 4% GV showed less potency of virucidal effect against SARS-CoV-2 compared with 4% WDV and 4% AA, although they should contain same kind and concentration of weak acid with similar pH. GV and WDV contain other organic components extracted from plants such as sugar, and these different components may influence the effects of GV and WDV. Our results demonstrate the potential antiviral properties of these edible vinegars, which can be used as sanitizers against enveloped viruses in common household.

Interestingly, our results showed that SARS-CoV-2 was comparatively stable against inactivation by acetic and oxalic acids under the same pH conditions when compared with FulV, although both viruses are enveloped. In particular, oxalic acid did not inactivate SARS-CoV-2, even at pH 2. These observations indicate that this virus is more stable under low pH conditions than other enveloped viruses. A previous study showed that SARS-CoV-2 was extremely stable over a wide pH range [27]. Wang et al. recently showed that oxalic acid exhibited inhibitory effects on combination to the receptor-binding domains of the SARS-CoV-2 pseudovirus and angiotensin-converting enzyme 2 [28]. Although our data showed no changes in infectious ability of SARS-CoV-2 after oxalic acid reactions, the elucidation of the inconsistent observations may provide evidence to reveal the factors affecting the stability of SARS-CoV-2 under acidic conditions.

Some coronaviruses, such as transmissible gastroenteritis virus [29] and porcine epidemic diarrhea virus [30], cause oral infections and intestinal diseases, such as diarrhea, suggesting that these coronaviruses are resistant to gastric acid. Therefore, coronaviruses potentially possess resistance to low pH among enveloped viruses. It would be interesting to reveal the mechanism and factors underlying the low pH stability of enveloped coronaviruses, which cause gastrointestinal infections. Further investigation of these factors will provide useful information for the development of more effective antiviral agents against coronaviruses, including SARS-CoV-2.

## Conclusions

When comparing acetic, oxalic, and citric acids, we observed that the inactivation efficacies of weak acids are different, even under the same pH conditions. These observations suggest that the virucidal effect of weak acids is not simply determined by pH and additional factors may also influence these effects. Acetic acid shows an effective virucidal reaction against enveloped viruses compared with oxalic and citric acids. Eligible vinegars, whose main component is acetic acid, may be potential sanitizers for some enveloped viruses, such as FulV in the domestic environment. In addition, our data imply that SARS-CoV-2 is comparatively stable against inactivation by weak acids. Further investigation to reveal the mechanism and factors of low pH stability of enveloped coronaviruses will provide useful information for the development of more effective antiviral agents against coronaviruses.

### Supplementary Information

**Additional file 1**. pH values in diluent reaction solutions of pH-dependent acids and 2%FBS DMEM for plaque assays.

**Additional file 2**. pH values in diluent reaction solutions of molarity concentration-dependent acids and 2%FBS DMEM for plaque assays.

**Additional file 3**. pH values in diluent reaction solutions of edible vinegar and DMEM for plaque assays.

**Additional file 4**. Quantification of cell viability in diluent reaction solutions of weak acids corresponding to pH value.

**Additional file 5**. Quantification of cell viability of diluent reaction solutions of vinegar corresponding to pH value.

## Data Availability

Not applicable.

## Abbreviations

FulV: Influenza virus
FCV: Feline calicivirus
DMEM: Dulbecco’s modified Eagle’s minimum essential medium
FBS: Fetal bovine serum
BSL: Biosafety level
BSA: Bovine serum albumin
pi: Post-infection
pfu: Plaque-forming units
WDV: White distilled vinegar
GV: Grain vinegar
NIID: National Institute of Infectious Diseases
FBS: Fetal bovine serum
NaOH: Sodium hydroxide
