# Supplementary material for: pH-dependent virucidal effects of weak acids against pathogenic viruses
Source: Trop Med Health. 2024 Jan 12;52:9. doi: 10.1186/s41182-023-00573-1 (PMC10785384; doi:10.1186/s41182-023-00573-1)
Supplement: Supplementary file 1 — Additional file 1. pH values in diluent reaction solutions of pH-dependent acids and 2%FBS DMEM for plaque assays. [file 41182_2023_573_MOESM1_ESM.docx]

Table S1. pH values in diluent reaction solutions of pH-dependent acids and

2%FBS DMEM for plaque assays

| Acid | pH | Diluents | | | | |
| --- | --- | --- | --- | --- | --- | --- |
|  |  | 1 | 0.1 | 0.01 | 0.001 | 0.0001 |
| Acetic acid | pH2 | 2.21 | 3.46 | 4.99 | 7.22 | 7.92 |
|  | pH4 | 4.18 | 5.21 | 7.27 | 7.95 | 7.95 |
|  | pH6 | 5.96 | 7.87 | 8.25 | 8.26 | 8.23 |
| Oxalic acid | pH2 | 1.92 | 5.93 | 7.93 | 8.32 | 8.27 |
|  | pH4 | 4.05 | 6.95 | 8.1 | 8.21 | 8.2 |
|  | pH6 | 6.94 | 8.27 | 8.25 | 8.22 | 8.25 |
| Citric acid | pH2 | 1.97 | 3.12 | 6.4 | 7.79 | 8.09 |
|  | pH4 | 4.13 | 4.45 | 6.06 | 7.51 | 8.09 |
|  | pH6 | 6.15 | 6.90 | 7.84 | 8.11 | 8.17 |

Reaction mixtures of weak acids (pH2, 4, 6) and 2%FBS DMEM (9:1) were followed to 10-fold serially dilution (1-0.0001) and pH of each diluent was measured.
