# Supplementary material for: pH-dependent virucidal effects of weak acids against pathogenic viruses
Source: Trop Med Health. 2024 Jan 12;52:9. doi: 10.1186/s41182-023-00573-1 (PMC10785384; doi:10.1186/s41182-023-00573-1)
Supplement: Supplementary file 2 — Additional file 2. pH values in diluent reaction solutions of molarity concentration-dependent acids and 2%FBS DMEM for plaque assays. [file 41182_2023_573_MOESM2_ESM.docx]

Table S2. pH values in diluent reaction solutions of molarity concentration-dependent acids and 2%FBS DMEM for plaque assays

| Acid | Molarity concentration | Diluents | | | | |
| --- | --- | --- | --- | --- | --- | --- |
|  |  | 1 | 0.1 | 0.01 | 0.001 | 0.0001 |
| Acetic acid | 1M | 2.60 | 4.46 | 7.00 | 8.15 | 8.03 |
|  | 0.1M | 3.28 | 6.87 | 7.90 | 8.01 | 7.96 |
|  | 0.01M | 4.47 | 8.00 | 8.13 | 8.16 | 8.11 |
|  | 0.001M | 7.19 | 8.16 | 8.18 | 8.13 | 8.13 |
| Citric acid | 1M | 1.59 | 2.91 | 6.24 | 7.70 | 7.99 |
|  | 0.1M | 2.28 | 6.27 | 7.80 | 8.07 | 8.04 |
|  | 0.01M | 3.34 | 7.72 | 8.08 | 8.13 | 8.11 |
|  | 0.001M | 6.68 | 8.10 | 8.15 | 8.09 | 8.09 |
| Oxalic acid | 1M | 1.03 | 1.61 | 6.16 | 7.58 | 7.87 |
|  | 0.1M | 1.49 | 6.26 | 7.66 | 7.97 | 7.91 |
|  | 0.01M | 2.33 | 7.63 | 7.97 | 7.93 | 7.80 |
|  | 0.001M | 6.63 | 8.04 | 8.03 | 8.03 | 7.98 |

Reaction mixtures of weak acids (1-0.001M) and 2%FBS DMEM (9:1) were followed to 10-fold serially dilution (1-0.0001) and pH of each diluent was measured.
