# Supplementary material for: pH-dependent virucidal effects of weak acids against pathogenic viruses
Source: Trop Med Health. 2024 Jan 12;52:9. doi: 10.1186/s41182-023-00573-1 (PMC10785384; doi:10.1186/s41182-023-00573-1)
Supplement: Supplementary file 3 — Additional file 3. pH values in diluent reaction solutions of edible vinegar and DMEM for plaque assays. [file 41182_2023_573_MOESM3_ESM.docx]

Table S3. pH values in diluent reaction solutions of edible vinegar and DMEM for plaque assays

| Acid | Percentage | Original pH | Diluents | | | | |
| --- | --- | --- | --- | --- | --- | --- | --- |
|  |  |  | 1 | 0.1 | 0.01 | 0.001 | 0.0001 |
| GV | 4% | 2.76 | 2.67 | 4.48 | 7.39 | 8.17 | 8.61 |
| WDV | 4% | 2.12 | 2.55 | 4.99 | 8.10 | 8.55 | 8.82 |
| AA | 4% | 2.23 | 2.54 | 4.76 | 7.61 | 8.17 | 8.77 |

pH in original vinegar (4%GV, 4% WDV, 4% AA was measured before mixing with 2%FBS DMEM. Reaction mixtures of vinegar (4%GV, 4% WDV, 4% AA) and 2%FBS DMEM (9:1) were followed to 10-fold serially dilution (1-0.0001) and pH of each diluent was measured.
