# Supplementary material for: pH-dependent virucidal effects of weak acids against pathogenic viruses
Source: Trop Med Health. 2024 Jan 12;52:9. doi: 10.1186/s41182-023-00573-1 (PMC10785384; doi:10.1186/s41182-023-00573-1)
Supplement: Supplementary file 4 — Additional file 4. Quantification of cell viability in diluent reaction solutions of weak acids corresponding to pH value. [file 41182_2023_573_MOESM4_ESM.docx]

Table S4. Quantification of cell viability in diluent reaction solutions of weak acids corresponding to pH value

| Acid |  |  | Diluents | | | | |
| --- | --- | --- | --- | --- | --- | --- | --- |
|  |  |  | 0.1 | 0.01 | 0.001 | 0.0001 |  |
| Acetic acid | pH |  | 3.46 | 4.99 | 7.22 | 7.92 |  |
|  | OD value | Vero E6/TMPRSS2 | 0.23* | 0.20* | 0.52 | 0.57 |  |
|  |  | CRFK | 0.17* | 0.34* | 0.63 | 0.54 |  |
|  |  | MDCK | 0.28* | 0.59* | 0.85 | 0.66 |  |
| Oxalic acid | pH |  | 5.93 | 7.93 | 8.32 | 8.27 |  |
|  | OD value | Vero E6/TMPRSS2 | 0.25* | 0.61 | 0.59 | 0.63 |  |
|  |  | CRFK | 0.31* | 0.70 | 0.72 | 0.82 |  |
|  |  | MDCK | 0.34* | 0.79 | 0.84 | 0.79 |  |
|  |  |  | 0.249* | 0.61 | 0.59 | 0.63 |  |
| Citric acid | pH |  | 3.12 | 6.4 | 7.79 | 8.09 |  |
|  | OD value | Vero E6/TMPRSS2 | 0.26* | 0.64 | 0.64 | 0.73 |  |
|  |  | CRFK | 0.23* | 0.58 | 0.69 | 0.81 |  |
|  |  | MDCK | 0.29* | 0.78 | 0.87 | 0.87 |  |

pH of each diluent (0.1-0.0001) of weak acids (acetic acid, oxalic acid and citric acid at pH 2) were measured. Cell viability of the diluents were tested by XTT cell viability assay. OD value: Absorbance_450nm_-Absorbance_655nm_. Control OD: Vero E6/TMPRSS2=0.6, CRFK=0.67, MDCK=0.77. “*” indicate cell damage.
