# Supplementary material for: pH-dependent virucidal effects of weak acids against pathogenic viruses
Source: Trop Med Health. 2024 Jan 12;52:9. doi: 10.1186/s41182-023-00573-1 (PMC10785384; doi:10.1186/s41182-023-00573-1)
Supplement: Supplementary file 5 — Additional file 5. Quantification of cell viability of diluent reaction solutions of vinegar corresponding to pH value. [file 41182_2023_573_MOESM5_ESM.docx]

Table S5. Quantification of cell viability of diluent reaction solutions of vinegar corresponding to pH value

| Acid | pH |  | Diluents | | | | |
| --- | --- | --- | --- | --- | --- | --- | --- |
|  |  |  | 0.1 | 0.01 | 0.001 | 0.0001 |  |
| 4% GV | pH |  | 4.48 | 7.39 | 8.17 | 8.61 |  |
|  | OD value | Vero E6/TMPRSS2 | 0.20* | 0.55 | 0.56 | 0.67 |  |
|  |  | CRFK | 0.27* | 0.66 | 0.75 | 0.75 |  |
|  |  | MDCK | 0.27* | 0.83 | 0.80 | 0.73 |  |
| 4% WDV | pH |  | 4.99 | 8.1 | 8.55 | 8.82 |  |
|  | OD value | Vero E6/TMPRSS2 | 0.19* | 0.72 | 0.59 | 0.76 |  |
|  |  | CRFK | 0.09* | 0.64 | 0.68 | 0.76 |  |
|  |  | MDCK | 0.25* | 0.92 | 0.90 | 0.86 |  |
| 4% AA | pH |  | 4.76 | 7.61 | 8.17 | 8.77 |  |
|  | OD value | Vero E6/TMPRSS2 | 0.17* | 0.54 | 0.59 | 0.62 |  |
|  |  | CRFK | 0.10* | 0.50 | 0.57 | 0.59 |  |
|  |  | MDCK | 0.27* | 0.84 | 0.88 | 0.86 |  |

pH of diluents (0.1-0.0001) of vinegar (4%GV, 4%WDV, 4%AA) were measured. Cell viability of the diluents were tested by XTT cell viability assay. OD value: Absorbance_450nm_-Absorbance_655nm_. Control OD: Vero E6/TMPRSS2=0.6, CRFK=0.67, MDCK=0.77. “*” indicated cell damage by diluents.
